# Assessment of the genetic parameters of soybean genotypes for precocity and productivity in the various cultivation conditions

**DOI:** 10.1016/j.heliyon.2024.e36135

**Published:** 2024-08-13

**Authors:** Gulden Kipshakbayeva, Meisam Zargar, Аiman Rysbekova, Inkar Ashirbekova, Zarina Tleulina, Bekzak Amantayev, Assemgul Kipshakbayeva, Aliya Baitelenova, Gani Stybayev, Meysam Soltani Nejad

**Affiliations:** a Department of Plant Production, Faculty of Agronomy, S. Seifullin Kazakh Agrotechnical University, Astana, 010000, Kazakhstan; b Department of Agrobiotechnology, Institute of Agriculture, RUDN University, 117198, Moscow, Russia; c Department of Plant Protection, Faculty of Agriculture, Ferdowsi University of Mashhad, Mashhad, Iran

**Keywords:** Soybean, Maturity time, Cultivar, Gene, Flowering time

## Abstract

Soybean (*Glycine max* [L.] Merr) plays a crucial role in the advancement of agriculture in Kazakhstan, serving as a promising food crop and feed source. The primary challenge in boosting soybean production in Northern Kazakhstan lies in the absence of soybean cultivars suited to the region's conditions. As such, the foremost focus of breeding initiatives should be on creating soybean varieties that possess both early maturity and satisfactory yield potential. The objective of this research was to assess the impact of maturity time (MT) on both the yield formation and the adaptive characteristics of soybean varieties from different origins. This evaluation was conducted by analyzing the outcomes of their testing under diverse cultivation conditions in the northern region of Kazakhstan. The soybean cultivars that were examined, originating from various sources, were classified into three primary groups. These groups varied in terms of their growing season duration as well as their yield levels. The way the alleles of the *E1–E4* flowering genes were spread out in the identified clusters showed that for soybean varieties where recessive alleles of the E1–E4 genes build up, the growing season usually shorter. Cultivars of Chinese, Russian, and domestic selections isolated as a result of the research were good initial material for use in local breeding programs. Within the framework of the clusters, an environmental assessment of soybean accessions was carried out, which made it possible to determine their degree of plasticity and, in general, their adaptive potential in the conditions of Northern Kazakhstan. The best cultivars were the Chinese selection ‘Dongnong 63’ and the Russian selection ‘SIBNIIK 315’. Hence, the present study successfully discovered soybean cultivars that possess exceptional adaptability and flexibility. These cultivars hold significant potential for cultivation and practical use in the specific environmental circumstances of northern Kazakhstan.

## 1 Introduction

Soybean (*Glycine max* [L.] Merr) is the primary source of protein, despite being classified as an oilseed [1]. It possesses a diverse array of applications in various industries such as food, feed, technical, and medical sectors [2]. Soybean seeds that are chosen from local sources typically have a protein content ranging from 39 % to 40 % and a fat content ranging from 19 % to 23 %. Soybean cultivation spans across approximately 100 million hectares of land globally and is practiced in the main agricultural regions of 90 countries [3]. The worldwide production of this crop surpasses 253 million tons. Soybean serves as the primary source for oil, meal, and feed production on a global scale, playing a substantial role in regional and national food initiatives [4,5]. According to USDA data, the main regions for soybean cultivation and production are as follows: Brazil (35–40 %), the USA (20 %), Argentina (12 %), China (12–13 %), and India (8 %), with Europe contributing around 2 % of the global soybean cultivation area. The average global yield is approximately 22.5 c/ha. The primary consumers are China, Brazil, the USA, and Argentina, collectively accounting for about 50 % of the worldwide soybean production. China is notably the top soybean importer, with an average annual production of 18.3 million tons and importing over 75 million tons annually [6,7]. The obstacles encountered in soybean cultivation in Kazakhstan are mainly due to the prevailing climatic conditions in the country. Northern Kazakhstan is recognized as a major region globally, as well as in Central Asia, for crop cultivation without irrigation [8]. Agricultural activities in Northern Kazakhstan are distinguished by ample land resources, fertile soils, and favorable natural and climatic conditions that support the production of premium quality grains [9]. The expansion of soybean cultivation into northern regions is being hindered by challenging environmental conditions marked by limited heat and moisture availability. Kazakhstan is located in a region characterized by a moderate climate, with a slight level of humidity. The sum of active temperatures in this area ranges from 2000 to 2200 °C, while the humidification coefficient falls between 0.8 and 1.0. Additionally, the air temperature in Kazakhstan exhibits a distribution pattern that varies with latitude. Growing crops in Northern Kazakhstan is also facing significant risks due to the threat of late spring and early autumn frosts [10]. When creating soybean cultivars specifically designed for the climate of Northern Kazakhstan, it is essential to take into account the restricted accumulation of effective temperatures throughout the growth and development phase, as well as the prolonged daylight hours [11]. Nevertheless, soybean cultivars exhibiting a neutral photoperiodic response possess the capability to initiate flowering and produce seeds at an earlier stage, even under extended daylight conditions [12]. The practical selection in agriculture involves addressing a set of economically beneficial traits and requires resolving paradoxical challenges associated with combining seemingly contradictory characteristics [13]. These challenges include reconciling early maturity with high productivity and balancing resistance to abiotic factors with the optimal capacity to realize productivity potential across diverse soil and climatic conditions. The initial phase of breeding work involves the examination of the initial material [14]. Maturity time plays a crucial role; as late maturation is considered a setback that is deemed unacceptable for agricultural producers. Cultivars of late-maturing groups respond to long daylight conditions by increasing the duration of the growing season; as a rule, they do not have time to ripen before the onset of stable low temperatures [[15], [16], [17]]. Background and humidification in the main phases of culture development, long daylight hours, etc. The lack of soybean varieties adapted to the conditions of Northern Kazakhstan is the main obstacle to increasing the acreage of crops. Therefore, the priority of breeding programs should be the breeding of soybean varieties that combine early maturity, acceptable yields and the quality of marketable products [18]. The variability in soybean production is significantly impacted by weather conditions, accounting for 60–80 % of the total factors influencing the yield [19]. The use of new highly productive adaptive soybean cultivars is an important low-cost technique that ensures successful introduction and a high economic effect when growing this crop. Despite the environmental plasticity of modern cultivars, soybeans can react negatively to changes in the external environment, reducing yields when moving to other regions [20]. Requirements for soybean cultivars vary depending on the location and their purpose of cultivation. They must respond well to fertilizers, with high rate of photosynthesis and rhizobial nitrogen fixation intensity, and suitable for mechanized cultivation and harvest [21,22]. Therefore, the main properties of the climatic condition of Kazakhstan are its sharp continentally and uneven distribution of natural precipitation. These climatic conditions of Northern Kazakhstan, favors soybean cultivars with a shorter maturity time (86–98 days) and relatively high yields are important [23].

*E* loci in soybeans, known as *E*1-*E*12 loci, control the duration of maturity time and response to photoperiod. The molecular genetic mechanism of these genes is not entirely clear yet, but all these loci control the growing season *E*1, *E*2 [24], *E*3 [25], *E*4 [25], *E*5 [26], *E*6 [27], *E*7 [28], *E*8 [29] have been identified at the molecular level [30].

It was shown that *E*1-*E*4 loci play a key role in the adaptation of soybean cultivars to different latitudes and are involved in the regulation of both pre-flowering and post-flowering growth of plants under different photoperiod lengths [[31], [32], [33], [34]].

The *E*1 gene extends the vegetative phase (sprouting - flowering) by 19–23 days, both with a 16-h day and with a longer day, but does not influence the generative phase (flowering - maturing). Genotypes *E*1*e*3*e*4 (k-5839, 6391, 6654, and 601669) are late flowering gene but do not lengthen the maturity time when moved to the northern latitudes. Genes *E*3 and *E*4 determine reactions to photoperiod in the vegetative phase, which reaches 30 days with a 20-h day but is absent with a 16-h day or less [35,36]. Cultivars with these genes, for example, Harosoy (k-5687) and Clark (k-5615), practically do not ripen in northern latitudes. Recessive alleles of all these genes cause photoperiod neutrality. Lack of sensitivity to day length and early maturity are determined by alleles *e*3 (k-5769) and *e*4 (k-4878). For the *E*1, *E*2, *E*3, and E4 loci, the genes they encode and allele-determining nucleotide sequences have been determined [32,37,38]. The objectives of this study were to study the initial material of soybeans of various origins using different methods for breeding in the direction of early maturity and productivity of varieties and to identify adaptive forms for the conditions of Northern Kazakhstan.

## 2 Materials and methods

### 2.1 Tests condition

The study was conducted in 2021–2023 at the laboratory of the NJSC “Kazakh Agrotechnical University named after. S. Seifullina (71°01′E″50°39′N). The experiment was performed according to the All-Russian Institute of Plant Growing guidelines and the field experiment methodology [39].

### 2.2 Observations of soybean phenology

Phenological observations of soybean plant growth and development was done in accordance with the methodology described by Fehr et al. (1979) [40]. For each sample, 25 plants were selected, and an analysis was carried out according to the elements of the crop structure. Harvesting was carried out at the maturation of the soybean accessions.

### 2.3 Weather conditions

The technology of crop cultivation was zonal. Each soybean cultivar was harvested at harvest maturity stage. Weather conditions in terms of temperature had slight differences, and in terms of moisture availability differed significantly from the long-term average indicators. The atmospheric conditions for the period 2021–2023 were different, and the response of soybean cultivars of different origins to cultivation conditions was accordingly different. Atmospheric precipitation varied across the years of research, as did the temperature background with 2022 and 2023 as the driest years. An important period for obtaining high yields and carrying out the breeding process was at the flowering and maturity stages. In 2023 atmospheric conditions, there was a sharp increase in the temperature against atmospheric and soil drought; accordingly, the flowering phase and subsequent phases took place in critical conditions, which in turn affected the yield level and the maturity time of soybean cultivars. For accessions with extended period than normal, their relatively optimal condition is developed, but the maturation of these varieties comes much later. During the years of study, uneven precipitation and aridity in the initial period of development were reported.

### 2.4 Phenological stages of plants

The dates of the onset for phenological stages of development depended mainly on the genetic characteristics of the studied soybean cultivars. In Northern Kazakhstan, the atmospheric condition is relevant for the crop between growing stage development stage with the following stages as the main phases: emergence - flowering and flowering-maturation. According to our findings, the earlier group maturation period is up to 35 days, and the latter group maturation period is up to 38 days. Flowering - maturation stages should not exceed 55–65 days, later periods of passage of these stages do not contribute to rapid maturation and harvesting. The object of research was 90 soybean cultivars and lines of various ecological and geographical origins: domestic selection - 58; Russian - 10 and Chinese selection - 22.

### 2.5 Molecular genetic studies

Molecular genetic analysis was performed on fresh trifoliate leaves and DNA was isolated by CTAB according to Saghai Maroof et al. (1984) [41]. Double-distilled water, free from DNase and RNase, was used to dissolve the DNA isolates their concentration was determined with a UV–Vis spectrophotometer (NanoDrop 2000, Thermo Fisher Scientific, Waltham, MA, USA). PCR analysis was carried out to identify alleles at the *E*1, *E*2, *E*3 and *E*4 loci. DNA marker sequences for identifying *E*1, *E*2, *E*3 and *E*4 genes are listed in Table 1.

**Table 1:** Sequences of DNA markers for identification of alleles at the E1, E2, E3 and E4 loci.

Marker	Primer	Sequence (5′–3′)
E1_*Hinf*I	G33snpTaqcutF	TCAGATGAAAGGGAGCAGTGTCAAAAGAAGT
	G33snpTaqcutR1	TCCGATCTCATCACCTTTCC
*e*1-re_STS	E1M0535_FW	CCGTTTGATTGGTTTTTGGT
	E1P0305_RV	CCCTTCAGTTTCTGCAGCTC
	e1re_0188RV	GAGAAGACAAACAATTCGAG
*E*2_*Dra*I	SoyGI_dCAPaMs19300FW	GAAGCCCATCAGAGGCATGTCTTATT
	SoyGI_dCAPa19440RV	GAGGCAGAGCCAAAGCCTAT
*E*2_InDel	E2_15345FW	TGTTGATATTACATGCACATGCAT
	E2_15856RV	GGCAGTTTCACCTTCTTAGC
*E*3*E*4_Mix	E3_08420FW	TGGGTCTTCAGTTCAGTTGG
	E3_09908RV	CTAAGTCCGCCTCTGGTTTCAG
	E3Ha_1000RV	CGGTCAAGAGCCAACATGAG
	e3T_0716RV	GTCCTATACAATTCTTTACGACG
	PhyA2-for	AGACGTAGTGCTAGGGCTAT
	PhyA2-Rev/E4	GCATCTCGCATCACCAGATCA
	PhyA2-Rev/e4	GCTCATCCCTTCGAATTCAG

The PCR amplification was run using a VeritiPro™ Thermal Cycler (Applied Biosystems, Singapore) with the following program: denaturation at 94 °C for 5 min, 2.35 cycles of 20 s at 94 °C; primer annealing 30 s at 58 °C; synthesis 60 s at 72 °C3. Extension at 72 °C for 10 min.

The PCR products or digested fragments were separated by 10 % polyacrylamide gel. Electrophoresis was carried out at a voltage of 120 V–180 V for 1 h. The results of electrophoresis were visualized and photographed by using gel-documentation system (Vilber, Russia, 2010). Computation for determining plasticity (bi) and stability (σd2) were performed using the method by Eberhart et al. (1966) [42].

### 2.6 Statically analysis

A check on the research results for reliability was carried out using a multivariate analysis of variance method using Microsoft Excel software and the Statistic 10 software package. Differences in the results obtained are possible at a significance level of P ≤ 0.05 according to Student's test.

## 3 Results

### 3.1 Growing season index

The growing season plays a crucial role in the formation of the yield, adaptive and economic properties of the variety. The growing season of a variety is not a constant value; it varies both geographically and by year. The variability of the growing season from year to year is determined mainly by factors: temperature, precipitation and biological characteristics of cultivars. The duration of the planting season from sowing to flowering solely depends on the sum of average daily temperatures, duration of seed filling, sum of temperatures, and the soil and air conditions. Cool weather and low positive temperatures during the period of growth and development change the course of physiological and biochemical processes, delay the development and formation of plants, and cause an increase in the duration of the growing season. During years of study, the growing season indicator was entirely dependent on cultivation conditions and the genetic characteristics of soybean accessions. Fig. 2 (a - c) shows soybean accessions according to their origin and length of the maturity time.

### 3.2 Effect on the duration of the growing season

**Fig. 1:**
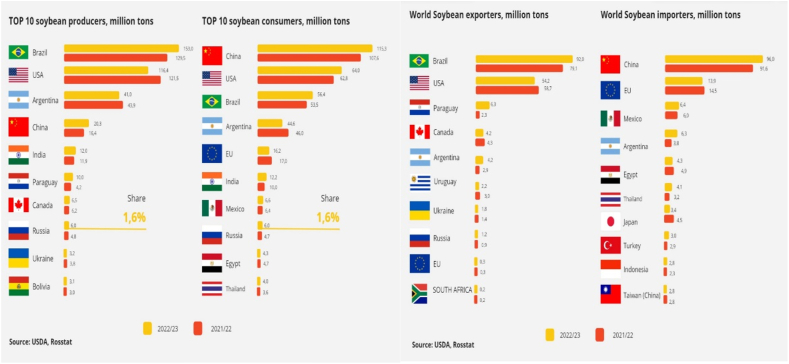
World soybean production, 2022–2023.

The length of the growing season, as well as the rhythm of development, is a powerful means of plant adaptation to environmental conditions, characterizing a variety or sample degree of maturation (Goncharov, 1993). The data shown in Fig. 1, presents the study on soybean varieties with difference in the duration of the growing season among groups and their origin. The term growing season refers to the time from germination to the end of the pod maturation, which coincides with the onset of full ripeness. During the “sprouting-flowering” period, the growth and development of vegetative organs occurs, contributing to the accumulation of total plant biomass.

**Fig. 2:**
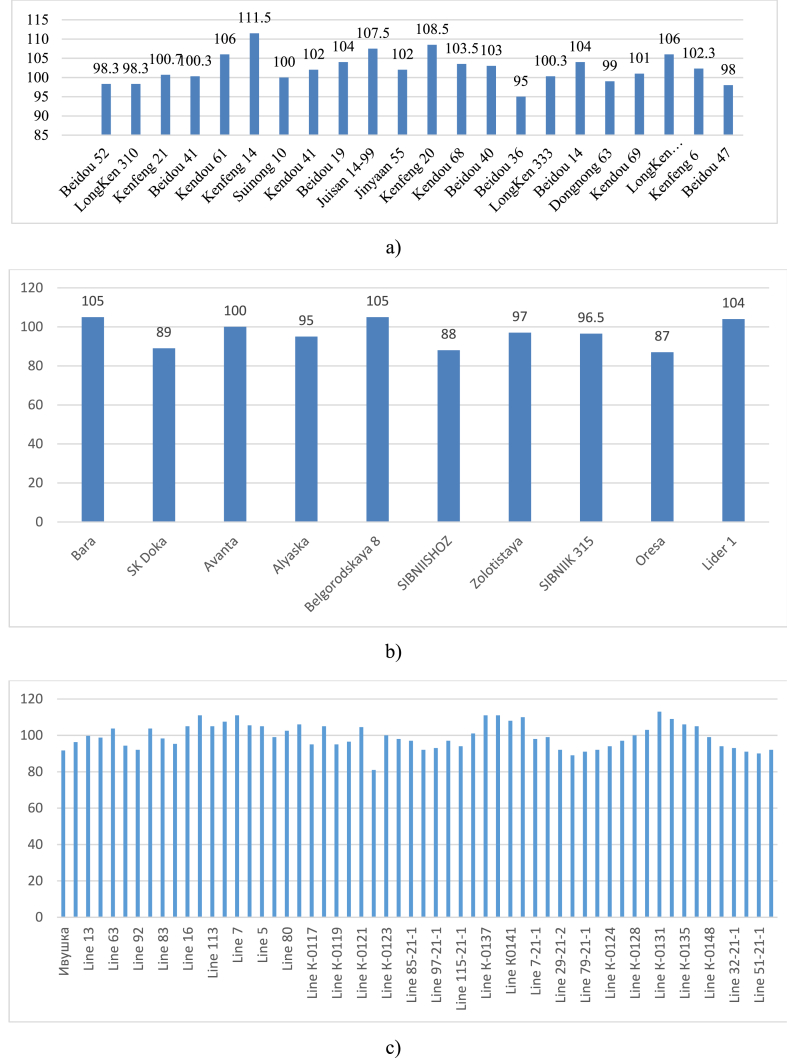
Length of the maturity time in the context of the studied soybean varieties and their origin. (a) accessions of Chinese selection, (b) accessions of Russian selection and (c) accessions of domestic selection.

To determine the effect on the duration of the growing season of variability and yield caused by varietal diversity, a two-factor analysis of variance was carried out; the results are presented in Table 2, Table 3. The results of the analysis of variance of data on the duration of the growing season, presented in Table 2, show that the options reflecting genotypic variability, variability caused by meteorological conditions (years), and the interaction of these two factors are reliable with high probability (P < 0.001). At the same time, the share of the influence of the variety was 55 %, the share of the influence of the duration of the growing season was 45 %, and at the 0.05 level the influence of varieties on the duration of the growing season was significant. The experimental error was 1.73, NSD -4.86.

**Table 2:** ANOVA of growing season affected seedlings – full maturing.

Source of Variation	SS	Df	MS	F	P-Value F critical	P-Value F critical
Between groups	4206.91	54.00	77.91	2.44	0.00	1.45
Within groups	3512.000	110.00	31.93			
Total	7718.91	164.00

**Table 3:** ANOVA of growing season affected yield level.

Source of Variation	*SS*	*Df*	*MS*	*F*	P-Value F critical	P-Value F critical
Between groups	402.43	54.00	7.45	0.77	0.86	1.45
Within groups	1067.72	110.00	9.71			
Total	1470.15	164.00				

### 3.3 Formation of the yield

The results of the analysis of variance on the formation of the yield level presented in Table 3 show a high share of the influence of cultivation conditions (73 %) and, accordingly, 27 % is the share of the influence of the variety. At the 0.05 level, the influence of varieties on the formation of this trait is not insignificant and is correspondingly low. The experimental error was 1.73 and the NSR was 4.84. The degree of realization of the genetic productivity potential of the studied soybean cultivars depends on a large number of exogenous factors, which are determined by cultivation conditions and are characterized by high variability, which entails significant variability in yield.

### 3.4 Genetically analysis

In soybeans, genetic control of flowering time has been used in classical breeding programs for many years and is important for the effective development of cultivars for relatively northern growing areas, predominantly.

The gene sequences responsible for the *E*1, *E*2, *E*3 and *E*4 loci and their flanking regions were analyzed in a soybean collection to identify the most suitable parental genotypes for use in local breeding programs. Accessions of the studied genotypes were divided into three geographically separate groups, and in each group the number of accessions turned out to be uneven. The collection included accessions from Chinese (Table 4), Russian (Table 5) and Kazakh selection (Table 6) (see Table 7).

**Table 4:** Identification of alleles in loci E1, E2, E3 and E4 of Chinese soybean accessions, 2023.

№	accessions	Alleles at loci			
		E1	E2	E3	E4
1	Beidou 52	Е1	e2-ns	Е3-Mi	Е4
2	LongKen 310	Е1	e2-ns	Е3-Mi	Е4
3	Kenfeng 21	Е1	e2-ns	Е3-Mi	Е4
4	Beidou 41	Е1	e2-ns	Е3-Mi	Е4
5	Kendou 61	Е1	e2-ns	Е3-Mi	Е4
6	Kenfeng 14	Е1	e2-ns	Е3-Mi	Е4
7	Suinong 10	Е1	e2-ns	Е3-Mi	Е4
8	Kendou 41	e1-as	e2-ns	Е3-Mi	е4-SORE-1
9	Beidou 19	Е1	e2-ns	Е3-Mi	Е4
10	Juisan 14-99	Е1	e2-ns	е3-tr	Е4
11	Jinyaan 55	Е1	e2-ns	Е3-Mi	Е4
12	Kenfeng 20	e1-as	E2-in	Е3-Mi	е4-SORE-1
13	Kendou 68	Е1	e2-ns	Е3-Mi	Е4
14	Beidou 40	Е1	e2-ns	Е3-Mi	Е4
15	Beidou 36	Е1	e2-ns	Е3-Mi	Е4
16	LongKen 333	Е1	e2-ns	Е3-Ha	Е4
17	Beidou 14	Е1	e2-ns	Е3-Mi	Е4
18	Dongnong 63	Е1	e2-ns	Е3-Mi	Е4
19	Kendou 69	e1-as	E2-in	Е3-Mi	Е4
20	LongKen 336/1	e1-as	E2-in	Е3-Ha	Е4
21	Kenfeng 6	e1-as	e2-ns	Е3-Mi	Е4
22	Beidou 47	Е1	e2-ns	Е3-Mi	Е4

**Table 5:** Identification of alleles at loci E1, E2, E3 and E4 of Russian soybean accessions, 2023.

№	accessions	Alleles at loci			
		E1	E2	E3	E4
1	Bara	e1-as	e2-ns	Е3-Mi	Е4
2	SK Doka	Е1	e2-ns	Е3-Mi	Е4
3	Avanta	Е1	e2-ns	Е3-Mi	Е4
4	Alyaska	e1-as	e2-ns	Е3-Ha	Е4
5	Belgorodskaya 8	e1-fs	e2-ns	Е3-Mi	е4-SORE-1
6	SIBNIISHOZ	Е1	e2-ns	Е3-Mi	е4-SORE-1
7	Zolotistaya	*Е*1	*e*2-*ns*	*Е*3-*Ha*	*Е*4
8	SIBNIIK 315	*Е*1	*e*2-*ns*	*Е*3-*Mi*	*е*4-*SORE-*1
9	Oresa	*Е*1	*e*2-*ns*	*Е*3-*Mi*	*Е*4
10	Lider 1	*Е*1	*e*2-*ns*	*Е*3-*Mi*	*е*4-*SORE-*1

**Table 6:** Identification of alleles in E1, E2, E3 and E4 loci of Kazakhstan soybean accessions, 2023.

№	accessions	Alleles at loci			
		E1	E2	E3	E4
1	Ивушка	Е1	e2-ns	Е3-Mi	Е4
2	Line 8	e1-as	e2-ns	Е3-Mi	Е4
3	Line 13	e1-as	e2-ns	Е3-Mi	Е4
4	Line 78	e1-fs	e2-ns	Е3-Mi	Е4
5	Line 63	Е1	E2-in	Е3-Mi	Е4
6	Line 33	Е1	E2-in	е3-tr	Е4
7	Line 92	Е1	e2-ns	е3-tr	Е4
8	Line 57	Е1	e2-ns	е3-tr	Е4
9	Line 83	Е1	e2-ns	е3-tr	Е4
10	Line 73	Е1	e2-ns	е3-tr	Е4
11	Line 16	e1-as	e2-ns	е3-tr	Е4
12	Line 75	e1-as	E2-dl	Е3-Mi	е4-SORE-1
13	Line 113	e1-as	e2-ns	Е3-Mi	Е4
14	Line 90	Е1	e2-ns	Е3-Mi	е4-SORE-1
15	Line 7	Е1	e2-ns	Е3-Mi	е4-SORE-1
16	Line 114	e1-as	e2-ns	Е3-Mi	е4-SORE-1
17	Line 5	Е1	E2-dl	Е3-Mi	Е4
18	Line 115	Е1	e2-ns	Е3-Mi	Е4
19	Line 80	Е1	e2-ns	Е3-Mi	е4-SORE-1
20	Line К-0115	Е1	e2-ns	NA	NA
21	Line К-0117	Е1	e2-ns	Е3-Mi	Е4
22	Line К-0118	Е1	e2-ns	Е3-Mi	е4-SORE-1
23	Line К-0119	e1-fs	e2-ns	Е3-Mi	е4-SORE-1
24	Line К-0120	Е1	e2-ns	Е3-Mi	Е4
25	Line К-0121	Е1	e2-ns	Е3-Mi	Е4
26	Line К-0122	Е1	E2-dl	NA	NA
27	Line К-0123	e1-fs	e2-ns	е3-tr	Е4
28	Line 51-21-3	Е1	e2-ns	Е3-Mi	Е4
29	Line 85-21-1	Е1	E2-dl	Е3-Mi	Е4
30	Line 85-21-2	e1-as	E2-dl	Е3-Mi	е4-SORE-1
31	Line 97-21-1	Е1	e2-ns	Е3-Mi	Е4
32	Line 110-21-1	e1-as	e2-ns	Е3-Ha	Е4
33	Line 115-21-1	e1-fs	e2-ns	Е3-Ha	Е4
34	Line К-0126	Е1	E2-dl	Е3-Ha	Е4
35	Line К-0137	e1-as	e2-ns	Е3-Ha	Е4
36	Line К-0140	e1-fs	e2-ns	Е3-Ha	Е4
37	Line К0141	Е1	e2-ns	Е3-Mi	е4-SORE-1
38	Line К-0142	Е1	e2-ns	Е3-Mi	е4-SORE-1
39	Line 7-21-1	Е1	e2-ns	Е3-Ha	Е4
40	Line 13-21-2	Е1	e2-ns	Е3-Mi	Е4
41	Line 29-21-2	Е1	e2-ns	Е3-Mi	Е4
42	Line 39-21-1	Е1	E2-dl	Е3-Mi	Е4
43	Line 79-21-1	Е1	e2-ns	Е3-Mi	Е4
44	Line 92-21-2	Е1	E2-dl	Е3-Mi	Е4
45	Line К-0124	*e*1-*as*	*e*2-*ns*	*Е*3-*Mi*	*Е*4
46	Line К-0127	*Е*1	*e*2-*ns*	*Е*3-*Ha*	*Е*4
47	Line К-0128	*Е*1	*e*2-*ns*	*Е*3-*Ha*	*Е*4
48	Line К-0129	*e*1-*fs*	*e*2-*ns*	*е*3-*tr*	*Е*4
49	Line К-0131	*Е*1	*e*2-*ns*	*Е*3-*Mi*	*Е*4
50	Line К-0134	*Е*1	*e*2-*ns*	*Е*3-*Mi*	*Е*4
51	Line К-0135	*Е*1	*e*2-*ns*	*Е*3-*Ha*	*Е*4
52	Line К-0136	*Е*1	*e*2-*ns*	*е*3-*tr*	*Е*4
53	Line К-0148	*Е*1	*e*2-*ns*	*Е*3-*Mi*	*е*4-*SORE-*1
54	Line 30-21-1	*Е*1	*e*2-*ns*	*Е*3-*Mi*	*Е*4
55	Line 32-21-1	*Е*1	*e*2-*ns*	*Е*3-*Mi*	*Е*4
56	Line 35-21-3	*Е*1	*e*2-*ns*	*Е*3-*Mi*	*Е*4
57	Line 51-21-1	*Е*1	*e*2-*ns*	*Е*3-*Mi*	*Е*4
58	Line 92-21-3	*Е*1	*e*2-*ns*	*Е*3-*Mi*	*е*4-*SORE-*1

**Table 7:** Productivity and indicators of stability and plasticity of soybean accessions (by clusters), 2021–2023.

No.	Accessions	Yield, c/ha			average	σ	σ^2^	CV, %	R	bi	σd^2^
		2021	2022	2023							
*Cluster 1*											
1	Line К-0122	1.6	2.1	8.5	4.1	3.14	9.86	77.3	6.9	0.9	73.3
2	Ivushka St	10.1	7.9	12.5	10.2	1.88	3.53	18.5	4.6	2.01	292.6
3	Line 92	6.8	5.1	12.7	8.2	3.26	10.6	39.7	7.6	1.71	213.5
4	Oresa	8.1	2.4	13.7	8.1	4.61	21.28	57.2	11.3	1.77	236.4
5	SK Doka	9.8	5.9	10.9	8.9	2.15	4.6	24.2	5.02	1.78	227.9
6	SIBNIISHOZ	3.6	8.9	5.3	5.9	2.21	4.88	37.2	3.4	1.02	110.3
*Cluster 2*											
1	Kendou 61	8.5	2.3	10.9	7.2	3.62	13.14	5.1	8.6	1.56	179.3
2	LongKen 336/1	8.6	5.2	5.6	6.5	1.52	2.3	23.5	3.4	1.22	121.4
3	Line К-0115	1.8	2.34	3.1	2.4	0.53	0.28	22.1	1.3	0.47	16.8
4	Line 114	8.9	5.8	11.3	8.7	2.25	5.07	25.9	5.5	1.75	219.5
5	Bara St	11.9	8.4	8.6	9.6	1.6	2.58	16.7	3.5	1.81	261.8
6	Line 16	4.6	12.1	7.3	8.01	3.1	9.62	38.8	7.5	1.37	202.9
7	Line 113	10.0	5.4	14.5	10.0	3.72	13.8	37.3	9.1	2.08	309.8
8	Line 5	3.7	4.8	12.4	7.0	3.87	14.96	55.5	8.7	1.47	175.6
9	Belgorodskaya 8	3.5	2.7	10.1	5.4	3.27	10.69	60.5	7.3	1.17	109.8
10	Line К-0121	1.2	1.8	13.4	5.5	5.62	31.52	102.7	12.2	1.28	171.2
11	Kendou 41	9.1	4.8	7.5	7.1	1.77	3.15	24.8	4.3	1.4	148.4
12	Jinyaan 55	8.7	7.3	9.7	8.6	0.98	0.97	11.5	2.4	1.66	203.6
13	Line 80	2.3	1.6	3.5	2.5	0.72	0.62	31.8	1.9	0.5	18.4
14	Beidou 40	6.8	4.9	11.8	7.8	2.91	8.47	37.2	6.9	1.62	191.4
15	Beidou 19	9.6	7.8	9.2	8.9	0.77	0.6	8.7	1.8	1.69	216.9
16	Beidou 14	9.4	7.6	11.6	9.5	1.63	2.68	17.2	4.0	1.87	256.2
17	Lider 1	6.5	2.5	10.4	6.5	3.23	10.4	49.87	7.9	1.39	143.0
18	Kendou 68	7.4	6.2	7.5	7.0	0.59	0.35	8.4	1.3	1.35	136.4
19	Line 63	10.5	4.5	12.4	9.1	3.37	11.3	36.9	7.9	1.9	259.5
20	Line 57	8.7	3.9	9.8	7.5	2.56	6.56	34.3	5.9	1.54	170.7
21	Line 75	9.8	2.3	5.6	6.1	3.07	9.42	52.0	7.5	1.19	130.1
22	Juisan 14-99	4.7	4.9	5.3	5.0	0.24	0.06	50.1	0.6	0.94	67.7
*Cluster 3*											
1	Dongnong 63	5.9	5.3	6.1	5.8	0.34	0.12	5.9	0.8	1.1	91.4
2	Line 115	3.9	3.6	14.8	7.4	5.21	27.15	70.1	11.2	1.64	227.8
3	Line 78	8.1	10.4	15.4	11.3	3.05	9.28	26.9	7.3	2.22	376.5
4	Beidou 47	4.7	10.1	7.9	7.6	2.22	4.92	29.3	5.4	1.35	171.2
5	LongKen 310	12.5	5.6	7.7	8.6	2.88	8.4	35.6	6.9	1.67	227.2
6	Beidou 52	12.4	5.3	3.3	7.0	3.9	15.24	55.8	9.1	1.28	179.8
7	Line 83	9.8	7.8	8.8	8.8	0.82	0.67	9.3	2.2	1.67	213.9
8	Beidou 36	10.1	6.1	6.8	7.7	1.74	3.04	22.8	6.1	1.46	170.0
9	Line 73	9.8	5.8	6.9	7.5	1.69	2.85	22.5	4.1	1.43	162.4
10	Line 33	7.6	7.9	6.1	7.2	0.79	0.62	10.9	1.8	1.3	143.6
11	Alyaska	9.1	5.5	8.3	7.6	1.54	2.38	20.2	3.6	1.5	166.3
12	Line К-0120	1.7	2.2	7.3	3.7	2.53	6.4	67.7	5.6	0.81	56.4
13	SIBNIIK 315	3.8	7.3	5.8	5.6	1.43	2.06	25.5	3.5	1.01	92.9
14	Line 8	11.9	4.3	11.9	9.4	3.58	12.83	38.3	7.6	1.94	275.9
15	Zolotistaya	5.9	10.7	6.6	7.7	2.11	4.48	27.4	4.8	1.34	176.3

When bi = 1 (or close to 1), the variety is considered low-plastic (does not respond to changes in environmental conditions); with bi > 1, the variety is highly plastic, intensive type (strongly responds to improved environmental conditions); when bi = 0 (or close to 0), the variety reacts weakly to changes in cultivation conditions (extensive type cultivar). The σd2 indicator characterizes the stability of the trait under various conditions: the lower the value of σd2, the more stable the trait.

Under long-day conditions, dominant alleles of *E* genes lengthen the maturity time, and recessive alleles of E genes, on the contrary, shorten it. Genotyping of soybean accessions for genes *E*1-*E*4 was carried out using DNA markers. To analyze the *E*1 gene, markers E1_HinfI and *e*1-re_STS were used, flanking the region of dominant and recessive alleles. As a result of PCR analysis of seven soybean samples, the presence of the e1-fs allele was detected, 17 samples had the e1-as allele, and the remaining 66 genotypes had the dominant *E*1 allele. The main product found in the *E*1 gene was 235 bp in size, 117 bp. and 80 bp, and in the e1-as allele was characterized by the presence of PCR products of 235 bp, 117 bp, 46 bp. and 33 bp sizes (Supplementary Fig. 1.

According to the marker *e*1-re_STS, the dominant *E*1 gene had a PCR product of 840 bp in size, while the recessive alleles *e*1-as and *e*1-fs had a PCR product of 841 bp in size (Supplementary Fig. 2).

Genotyping of the *E*2 gene in the soybean collection using the *E*2_*Dra*I marker allowed us to identify three types of alleles: *e*2-ns, *E*2-dl and *E*2-in. Of the entire collection, five genotypes had the *E*2-in allele, 8 genotypes had the *E*2-dl allele, the remaining 77 all samples had the e2-ns allele. PCR product of the dominant allele, 142 bp in size. not cleaved by endonuclease *Dra*I. The recessive allele *e*2 has a *Dra*I restriction site due to the A→T nucleotide substitution. As a result of hydrolysis of the PCR product, two DNA fragments of 115 and 27 bp in length are formed. Supplementary Fig. 3 shows the presence of the recessive allele *e*2 in the studied soybean samples.

The use of the *E*2_InDel marker to identify alleles of the *E*2 locus allowed us to obtain products Using this marker, the dominant allele *E*2-*dl* was identified in accessions Kenfeng 20, Kendou 69, LongKen, 336/1, Line 63 and Line 33, while the E2-in allele was identified in accessions Line 75, Line 5, Line K-0122, Line K −0126, lines 39-21-1, 85-21-1, 85-21-2 and 92-21-2. Based on the results of the analysis, alleles *E*3-Mi, *E*3-Ha and e3-tr were identified in the *E*3 locus. The varieties Juisan 14–99, Line 33, Line 92, Line 57, Line 83, Line 73, Line 16, Line K-0123, Line K-0129 and K-0136 had the *e*3*-tr* allele, genotypes LongKen 333, LongKen 336/1, Alyaska, Line K-0124, Line K-0127, Line K-0128, Line K-0136, Line K-0137, Line K-0140, Zolotistaya, Line 7, Line 110 and Line 115 had the *E*3-Ha allele, the rest *E*3-Mi. For the *E*4 locus, two types of alleles were identified among 90 soybean accessions: *E*4 and *e*4-SORE-1 (Supplementary Fig. 5), 72 and 18 genotypes, respectively. With a length of 544 and 512 bp. (Supplementary Fig. 4).

**Fig. 3:**
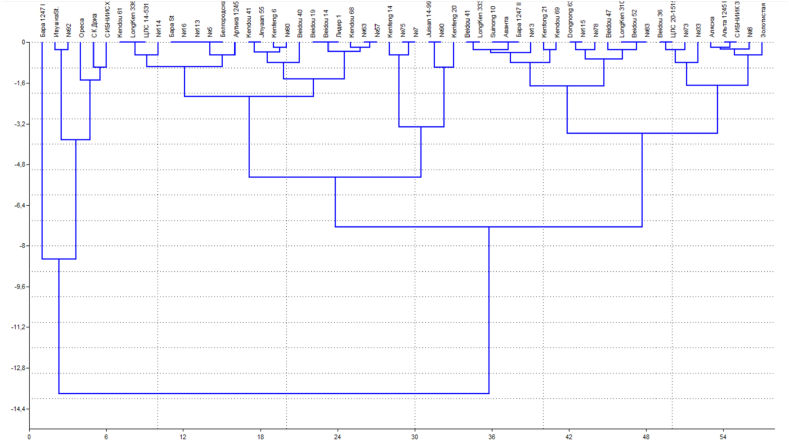
Dendrogram of cluster analysis of soybean varieties of different origins according to the range of the growing season, cf. for 2021–2023.

The *E*3 - *E*4_Mix marker made it possible to determine the dominant and recessive allele of the E4 locus by the presence of PCR products of 1229 and 837 bp in length. respectively. Using this marker, *E*4 and *e*4-SORE-1 allele types were identified in the soybean collection. In genotypes Line K-015 and Bara 1247 I, not a single allele was identified at the *E*3 and *E*4 loci. In order to identify promising soybean samples based on the main characteristic - the length of the growing season, cluster analysis was used (Fig. 3).

**Fig. 4:**
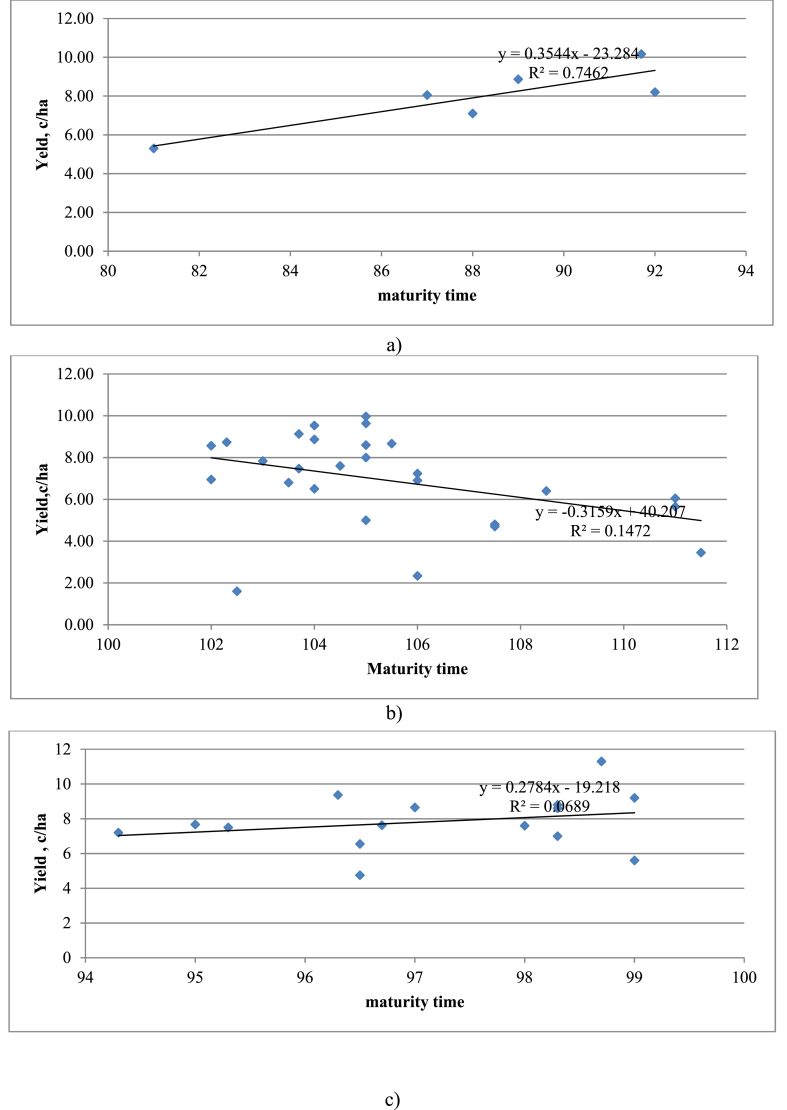
Regression of the yield of soybean accessions on the duration of their growing seasons in the context of the studied clusters. a) 1st cluster, b) 2nd cluster, c) 3rd cluster.

Cluster analysis made it possible to group the most similar and similar soybean accessions, regardless of their origin. According to Fig. 3, Fig. 4 main clusters were formed. The effectiveness of the classification was confirmed by the results of analysis of variance. The first cluster united six, the second – 27, and the third – 16 genotypes.

*1 cluster* – Line K-0122 (Kazakhstan), Ivushka (Kazakhstan), Line 92 (Kazakhstan), Oresa (Russia), SK Doka (Russia), SIBNIISKHOZ (Russia);

*Cluster 2* – Kendou 61 (China), LongKen 336/1 (China), Line K-0115 (Kazakhstan), Line 114 (Kazakhstan), Bara (Russia), Line 16 (Kazakhstan), Line 113 (Kazakhstan), Line 5 (Kazakhstan), Belgorodskaya 8 (Russia), Line K-0121 (Kazakhstan), Kendou 41 (China), Jinyaan 55 (China), Kenfeng 6 (China), Line 80 (Kazakhstan), Beidou 40 (Kazakhstan), Beidou 19 (Kazakhstan), Beidou 14 (Kazakhstan), Lider 1, Line 63 (Kazakhstan), Line 57 (Kazakhstan), Kendou 68 (Kazakhstan), Kenfeng 14 (Kazakhstan), Line 75 (Kazakhstan), Line 7 (Kazakhstan), Juisan 14–99 (China), Line 90 (Kazakhstan) and Kenfeng 20 (China);

*Cluster 3* – Dongnong 63 (China), Line 115 (Kazakhstan), Line 78 (Kazakhstan), Beidou 47 (China), LongKen 310 (China), Beidou 52 (China), Line 83 (Kazakhstan), Beidou 36 (China), Line K-0117 (Kazakhstan), Line 73 (Kazakhstan), Line 33 (Kazakhstan), Alyaska (Russia), Line K-0120 (Kazakhstan), SIBNIIK 315 (Russia), Line 8 (Kazakhstan) and Zolotistaya (Russia). It is needed to distinguish 2 and 3 clusters, where accessions of Chinese and domestic selection with deterministic maturity time 87–103 days are grouped. Using cluster analysis, purposefully select breeding material adapted to the conditions of the north of Kazakhstan. A study of the level of yield formation in different years of research shows that a strong negative and weak correlation is formed between yield and the duration of the maturity time in different years (r = −0.65 … 0.45). In our studies, the nature of this connection in the context of the formed clusters is as follows (Fig. 4).

The data presented in the diagrams proves a high relationship between the level of yield depending on the maturity time (in particular, depending on the slope of the regression line). Calculations to determine plasticity (bi) and stability (σd2) in the context of 3 clusters showed the following. It is believed that varieties with a plasticity index bi > 1 are more valuable. This requirement is met by all studied soybean accessions, regardless of the cluster. The most responsive to improving cultivation conditions in the north of Kazakhstan were the plastic varieties: in the 1st cluster - Ivushka (bi = 2.01), in the 2nd cluster - Line 113 (bi = 2.08), in the 3rd cluster – Line 78 (bi = 2.22) and Line 8 (bi = 1.94). All selected varieties belong to domestic selection and can be used in practical breeding as sources of plasticity in terms of yield and length of the growing season. The most valuable are soybean varieties with bi > 1, σd2 ≤ 1. These varieties can be considered highly stable (Table 6).

However, during the years of research it was not possible to isolate such accessions; the stability indicator varied widely. This conclusion was also proven by analysis of variance, where the formation of the studied indicators depended entirely on cultivation conditions (Table 2). The accessions with the most optimal values were Dongnong 63 (bi = 1.1, σd^2^ = 91.4) and SIBNIIK 315 (bi = 1.01, σd^2^ = 92.9) accessions from the 3rd cluster. As can be seen from the data in Table 3, had an increase in the bi index leads to a strong increase in the σd^2^ index. From this we must conclude that it is very important to carefully select the starting material for practical selection, both in the direction of increasing productivity and creating more early maturing forms. The coefficient of variation (CV%) for the accessions of the 2nd cluster was very high; all these varieties were characterized by high yield variability - more than 32 %. In accessions of the 1st cluster, yield variability was weak (less than 18 %). According to the level of optimal manifestation of the indicator, accessions of domestic selection were identified as line 92 (CV = 39.7), line 16 (CV = 38.8), line 113 (CV = 37.3), line 63 (CV = 36.9) and line 57 (CV = 34.3). A variety sample of the Russian selection SIBNIISKHOZ (CV = 37.2) and accessions of the Chinese selection Beidou 40 (CV = 37.2) and LongKen 310 (CV = 35.6). It should be noted that most of the selected soybean accessions belonged to cluster 2.

## 4 Discussion

The early maturity of soybean cultivars is one of the main factors that determine the possibility of cultivating soybean crops in the conditions of Northern Kazakhstan [43]. The growing season of soybean cultivars according to the Western European and North American classification is divided into five groups depending on the sum of active temperatures above 10 °C [44]. Plants in group “000” need 1700–2000 °C for normal growth and development; “00” needs 2001–2400 °C; “0” needs 2401–2600 °C; “I” needs 2601–2800 °C; and “II” needs 2801–3000 °C [45]. In this study, the Duration of the maturity time of accession was 81–111 days which is in accordance of UN FAO classification of maturity time, of ultra-early maturing (000) (75–80 days), very early maturing (00) (81–90 days), early maturing (0) (91–110 days), early maturing (medium-early maturing) (I) (111–120 days), medium-maturing (II) (121–130 days), medium-late (131–150), late-maturing (151–160), very late-maturing (151–170), exclusively late-maturing (>170). Depending on the cluster, the distribution of accessions was determined by the length of the growing season and by their yield level. The length of the growing season of the studied accessions in cluster 1 varied from 81 to 92 days (11.5 % of all studied material), respectively. In this cluster, there was a relatively low yield of 5.30–8.87 c/ha^−1^. Cluster 2 is distinguished by the highest content of the studied soybean accessions (59.6 %). This cluster contains accessions with a long growing season of 108.5–111 days and a yield of up to 9.97 c/ha^−1^. This cluster includes accessions of domestic and Chinese selection. In the 3rd cluster, 28.9 % of the studied accessions are located, with varying lengths of the growing season of 95–99 days and a relatively high yield of up to 11.3 c/ha^−1^. A different focus of selection is noted here; accessions from Russian, Chinese, and domestic selections were included. A feature of this cluster is the absence of strong variation in both the length of the growing season and yield. Our research results indicated that the length of the growing season was the main limiting factor in achieving high crop yields under conditions of a short frost-free growth period.

The research led to the identification of several early maturing cultivars that exhibit both early maturation and high productivity. These cultivars were found in the 1st and 2nd clusters, and their genotypic variability was confirmed through variance analysis results. It was revealed that the formation and duration of individual periods and the growing season as a whole are influenced not only by the biological characteristics of the varieties but also by the meteorological conditions of the growing season [46]. The strong variability in the length of the growing season (CV = 13.1–64.2 %) indicates the importance of the direction of selection to increase the plasticity of new varieties and their stability over the years. Soybean is a photoperiodic, highly sensitive, short-day plant. Deviations of photoperiods from the biological optimum of soybeans have a noticeable impact on changes in the growing season, plant height and productivity [47,48]. The high responsiveness of soybeans to day length is dictated by the epistatic interaction involving both dominant and recessive alleles of genes associated with photoperiodic sensitivity [49]. Therefore, research on soybean varieties specially to advance their cultivation range to the Northern Kazakhstan, it is necessary to identify the genetic determinants of their sensitivity/insensitivity to photoperiod [50]. Allelic variation in the E1 gene includes a single nucleotide polymorphism (SNP) at nucleotide 44 resulting in an arginine to threonine missense mutation (*e*1-*as*), a single base deletion (adenine) at nucleotide 49 resulting in a premature stop codon at nucleotide 124 (*e*1-*fs*) and a null allele (e1-nl), in which the entire *E*1 gene was deleted [38]. The results of PCR analysis identified the recessive *e*1-*as* allele in 5 genotypes of Chinese selection (Kendou 4, Kenfeng 20, Kendou 69, LongKen 336/1, Kenfeng 6), in 2 genotypes the e1-*as* allele, in 1 genotype *e*1-*fs* the Russian allele, and in 10 accessions the e1-as allele and 6 accessions the *e*1-*fs* allele of Kazakhstan selection. At the same time, the frequency of the recessive allele for the *E*1 gene in the Chinese selection collection is 22.7 %, Russian 30 % and Kazakh 27 %. It is known that the dominant alleles *E*1 and *E*2, as well as the *e*1-*as* allele in the *E*1 gene, delay the time of maturation, while the recessive alleles *E*3 and *E*4 provide insensitivity to photoperiod length. According to the results of molecular analysis of the E2 gene, 19 accessions of Chinese, 10 accessions of Russian and 48 accessions of Kazakh selection had the *e*2-*ns* allele. In studies by Xu M. et al. It has been shown that recessive alleles of the *E*1–*E*4 genes are the result of mutations (frame shifts, nonsynonymous substitutions, deletions) leading to protein dysfunction, which leads to insensitivity to photoperiod [34]. Genes *E*3 and E4 encode phytochrome A: GmPHYA3 and GMPHYA2, respectively. The collection of Russian selection was dominant in the *E*3 gene; *E*3-Mi and *E*3-*Ha* alleles were found in all genotypes; similar data were obtained from the collection of Chinese selection, with the exception of the Juisan 14–99 genotype, which had a recessive allele *E*3-*tr.* From the Kazakhstan selection, 9 accessions were characterized by the presence of the *e3*-t*r* allele: Line 33, Line 92, Line 57, Line 83, Line 73, Line 16, Line K-0123, Line K-0129 and Line K-0136, the remaining genotypes had dominant alleles E3-*Mi* and *E*3-Ha. For the *E*4 gene, the presence of the recessive allele *e*4-SORE-12 distinguished 2 genotypes Kendou 41 and Kenfeng 20 of Chinese selection, 4 varieties: Belgorodskaya 8, SIBNIISKHOZ, SIBNIIK 315 and Leader 1 of the Russian selection, as well as 13 samples of domestic selection, the frequency of the recessive allele was 9 %, 40 % and 22 % respectively. Thus, in our studies, only 8 combinations of alleles of the *E*1–*E*4 genes were identified: 1 - *e*2-*ns*; 2 - *e*1-*as*; 3 - *e*1-*fs*; 4 - *E*2-in; 5 - *E*2-dl; 6 - *e*3-tr; 7 - *E*3-Mi; 8 - *e*4-*SORE*-1. The data obtained indicate that the majority of varieties of Chinese selection are characterized by the presence of dominant alleles for the *E*1–*E*4 genes, while a significant part of the samples of Russian and Kazakh selection, on the contrary, have recessive alleles for the *E*1–*E*4 genes.

Varieties that exhibit a significant increase in their regression level under more favorable conditions are particularly intriguing. This characteristic signifies their high responsiveness to improved cultivation conditions and is accompanied by a modest decline under more challenging circumstances. This response pattern in the studied material is indicative of genotypes that differ in the degree of the studied traits under unfavorable cultivation conditions. Based on the results of environmental plasticity, the varieties Dongnong 63 (bi = 1.1, σd^2^ = 91.4) and SIBNIIK 315 (bi = 1.01, σd^2^ = 92.9) were identified from the 3rd cluster. Employing gene identification methods in classical breeding holds the promise of unveiling the mechanisms governing the length of the growing season, flowering timing, and ultimately, the development of high yields in the specific conditions of northern Kazakhstan.

## 5 Conclusions

In the course of the research, molecular markers for various alleles of the E1–E4 genes, responsible for photoperiod sensitivity and maturity time, were examined. The PCR analysis revealed a high frequency of dominant alleles at the E1, E2, and E4 loci in this soybean collection. Notably, for the E2 locus, the majority of the studied cultivars harbored the recessive e2-ns allele, which appears to be the primary contributor to the reduction in maturation time. These findings underscore the feasibility and importance of enhancing breeding programs to develop early maturing soybean varieties. The tested set of molecular markers can be employed in breeding efforts for soybean cultivars based on their sensitivity to photoperiod and maturity time, crucial factors influencing soybean productivity, especially in temperate climates, which are atypical for its cultivation.

## Data availability

Data will be made available on request.

## CRediT authorship contribution statement

**Gulden Kipshakbayeva:** Formal analysis, Data curation, Conceptualization. **Meisam Zargar:** Methodology, Formal analysis. **Аiman Rysbekova:** Writing – review & editing, Writing – original draft, Supervision. **Inkar Ashirbekova:** Project administration. **Zarina Tleulina:** Supervision, Project administration. **Bekzak Amantayev:** Writing – original draft, Supervision. **Assemgul Kipshakbayeva:** Writing – original draft, Validation. **Aliya Baitelenova:** Writing – original draft. **Gani Stybayev:** Writing – review & editing. **Meysam Soltani Nejad:** Methodology, Funding acquisition.

## Declaration of competing interest

The authors declare that they have no known competing financial interests or personal relationships that could have appeared to influence the work reported in this paper.

## References

[1] Ramya D., Sujatha P., Raghavendra K., Keshavulu K., Ramesh T., Radhika K. (2024). Antioxidants and polymer coating for soybean [*Glycine max* (L.) Merr.] seed longevity enhancement. Ind. Crops Prod..

[2] Ren H., Zhao K., Zhang C., Lamlom S.F., Liu X., Wang X., Zhang F., Yuan R., Gao Y., Cao B., Zhang B. (2024). Genetic analysis and QTL mapping of seed hardness trait in a soybean (*Glycine max*) recombinant inbred line (RIL) population. Gene.

[3] Abugaliyeva A.I., Didorenko S.V. (2016). Genetic diversity of soybean cultivars belonging to different ripeness groups with regard to performance and quality. Vavilov Journal of Genetics and Breeding..

[4] Medic J., Atkinson C., Hurburgh C.R. (2014). Current knowledge in soybean composition. JAOCS (J. Am. Oil Chem. Soc.).

[5] Hartman G.L., West E.D., Herman T.K. (2011). Crops that feed the World 2. Soybean—worldwide production, use, and constraints caused by pathogens and pests. Food Sec.

[6] Bukowski M.R., Goslee S.C. (2024). Climate-based variability in the essential fatty acid composition of soybean oil. Am J Clin.

[7] Su Q., Chen L., Cai Y., Wang L., Chen Y., Zhang J., Liu L., Zhang Y., Yuan S., Gao Y., Sun S., Han T., Hou W. (2023). The *FLOWERING LOCUS T 5b* positively regulates photoperiodic flowering and improves the geographical adaptation of soybean. Plant Cell Environ..

[8] Kienzler K.M., Lamers J.P.A., McDonald A., Mirzabaev A., Ibragimov N., Egamberdiev O., Ruzibaev E., Akramkhanov A. (2012). Conservation agriculture in Central Asia—what do we know and where do we go from here?. Field Crops Res..

[9] (2020). Land desertification and its influencing factors in Kazakhstan. J. Arid Environ..

[10] Wang D., Gao G., Li R., Toktarbek Shynggys, Jiakula Nueryia, Feng Y. (2022). Limiting factors and environmental adaptability for staple crops in Kazakhstan. Sustainability.

[11] Rau A., Koibakova Yelzaveta, Nurlan Balgabayev, Nabiollina Madina, Kurmanbek Zhanymhan, Issakov Yerlan, Zhu K. (2023). Lóránt dénes dávid, increase in productivity of chestnut soils on irrigated lands of northern and Central Kazakhstan. Land.

[12] Digrado A., Montes C.M., Baxter I., Ainsworth E.A. (2024). Seed quality under elevated CO_2_ differs in soybean cultivars with contrasting yield responses. Global Change Biol..

[13] Naserzadeh Y., Kartoolinejad D., Mahmoudi N., Zargar M., Pakina E., Heydari M., Astarkhanova T., Kavhiza N.J. (2018). Nine strains of *Pseudomonas fluorescens* and *P. putida*: effects on growth indices, seed and yield production of *Carthamus tinctorius* L. Research on Crops.

[14] Zargar M., Pakina E. (2014). Reduced rates of herbicide combined with biological components for suppressing weeds in wheat fields of Moscow, Russia. Res. Crops.

[15] Burlutskiy V.A., Peliy A.F., Borodina E.S., Diop A., Batygin A.S., Zargar M., Plushchikov V.G. (2020). Efficiency of advanced sprayers for nutrient and pesticide application under precision cultivation of spring rapeseed (*Brassica napus*). Research on Crops.

[16] Setiyono T.D., Weiss A., Specht J., Bastidas A.M., Cassman K.G., Dobermann A. (2007). Understanding and modeling the effect of temperature and daylength on soybean phenology under high-yield conditions. Field Crops Res..

[17] Karges K., Bellingrath-Kimura S.D., Watson C.A., Stoddard F.L., Halwani M., Reckling M. (2022). Agro-economic prospects for expanding soybean production beyond its current northerly limit in Europe. Eur. J. Agron..

[18] Zatybekov A., Yermagambetova M., Genievskaya Y., Didorenko S., Abugalieva S. (2023). Genetic diversity analysis of soybean collection using simple sequence repeat markers. Plants.

[19] Novikova L.Yu, Bulakh P.P., Nekrasov A.Yu, Seferova I.V. (2020). Soybean response to weather and climate conditions in the krasnodar and primorye territories of Russia over the past decades. Agronomy.

[20] Suleimenova N., Kalykov D., Makhamedova B., Oshakbaieva Z., Abildayev Y. (2021). A resource conservation technology for adapting argroecosystems to the new natural conditions of a warming climate in south-eastern Kazakhstan. Online J. Biol. Sci..

[21] Zhai H., Lü S., Wang Y., Chen X., Ren H., Yang J., Cheng W., Zong C., Gu H., Qiu H., Wu H., Zhang X., Cui T., Xia Z. (2014). Allelic variations at four major maturity E genes and transcriptional abundance of the E1 gene are associated with flowering time and maturity of soybean cultivars. PLoS One.

[22] Pliushchikov V., Bayat M., Zargar M., Akhrarov M., Orujov E., Hassan N.S. (2019). Common lambsquarters response to the ALS inhibitor herbicides. Res. Crops.

[23] Jiang B., Nan H., Gao Y., Tang L., Yue Y., Lu S., Ma L., Cao D., Sun S., Wang J., Wu C., Yuan X., Hou W., Kong F., Han T., Liu B. (2014). Allelic combinations of soybean maturity loci E1, E2, E3 and E4 result in diversity of maturity and adaptation to different latitudes. PLoS One.

[24] Bernard R.L. (1971). Two major genes for time of flowering and maturity in soybeans ^1^. Crop Sci..

[25] Buzzell R.I. (1971). Inheritance of a soybean flowering response to fluorescent-daylength conditions. Can. J. Genet. Cytol..

[26] McBlain B.A., Bernard R.L. (1987). A new gene affecting the time of flowering and maturity in soybeans. J. Hered..

[27] Bonato E.R. (1999). Natal Antônio Vello, E6, a dominant gene conditioning early flowering and maturity in soybeans. Genet. Mol. Biol..

[28] Cober E.R., Voldeng H.D. (2001). A new soybean maturity and photoperiod‐sensitivity locus linked to E1 and T. Crop Sci..

[29] Cober E.R., Molnar S.J., Charette M., Voldeng H.D. (2010). A new locus for early maturity in soybean. Crop Sci..

[30] Copley T., Marc-Olivier Duceppe, O'Donoughue L.S. (2018). Identification of novel loci associated with maturity and yield traits in early maturity soybean plant introduction lines. BMC Genom..

[31] Liu B., Kanazawa A., Matsumura H., Takahashi R., Harada K. (2008).

[32] Watanabe S., Hideshima R., Xia Z., Tsubokura Y., Sato S., Nakamoto Y., Yamanaka N., Takahashi R., Ishimoto M., Anai T., Tabata S., Harada K. (2009). Map-based cloning of the gene associated with the soybean maturity locus E3. Genetics.

[33] Watanabe S., Xia Z., Hideshima R., Tsubokura Y., Sato S., Yamanaka N., Takahashi R., Anai T., Tabata S., Kitamura K., Harada K. (2011). A map-based cloning strategy employing a residual heterozygous line reveals that theGIGANTEAGene is involved in soybean maturity and flowering. Genetics.

[34] Xu M., Xu Z., Liu B., Kong F., Tsubokura Y., Watanabe S., Xia Z., Harada K., Kanazawa A., Yamada T., Abe J. (2013). Genetic variation in four maturity genes affects photoperiod insensitivity and PHYA-regulated post-flowering responses of soybean. BMC Plant Biol..

[35] Buzzel R., Voldeng H. (1980).

[36] Tsubokura Y., Watanabe S., Xia Z., Kanamori H., Yamagata H., Kaga A., Katayose Y., Abe J., Ishimoto M., Harada K. (2013). Natural variation in the genes responsible for maturity loci E1, E2, E3 and E4 in soybean. Ann. Bot..

[37] Liu L., Song W., Wang L., Sun X., Qi Y., Wu T., Sun S., Jiang B., Wu C., Hou W., Ni Z., Han T. (2020). Allele combinations of maturity genes E1-E4 affect adaptation of soybean to diverse geographic regions and farming systems in China. PLoS One.

[38] Xia Z., Watanabe S., Yamada T., Tsubokura Y., Nakashima H., Zhai H., Anai T., Sato S., Yamazaki T., Lu S., Wu H., Tabata S., Harada K. (2012). Positional cloning and characterization reveal the molecular basis for soybean maturity locus E1 that regulates photoperiodic flowering. Proc. Natl. Acad. Sci. USA.

[39] Vishnyakova M.A., Seferova L.V., Buravtseva T.V., Burlyaeva M.O., Semenova E.V., Filipenko G.L., Drugova E.V. (2018).

[40] Fehr W.R., Cavines C.E. (1979).

[41] Saghai-Maroof M.A., Soliman K.M., Jorgensen R.A., Allard R.W. (1984). Ribosomal DNA spacer-length polymorphisms in barley: mendelian inheritance, chromosomal location, and population dynamics. Proc. Natl. Acad. Sci. USA.

[42] Eberhart S.A., Russell W.A. (1966). Stability parameters for comparing varieties 1. Crop Sci..

[43] Yerzhebayeva Raushan, Didorenko S., Amangeldiyeva Aigul, Daniyarova Aliya, Mazkirat Shynar, Zinchenko Alyona, Shavrukov Y. (2023). Marker-assisted selection for early maturing E loci in soybean yielded prospective breeding lines for high latitudes of northern Kazakhstan. Biomolecules.

[44] Grassini P., Cafaro La Menza N., Rattalino Edreira J.I., Monzón J.P., Tenorio F.A., Specht J.E. (2021). Soybean, crop physiology case histories for major crops.

[45] Rahmadina None, Nurwahyuni Isnaini, Elimasni None, Sofiah Hanafiah Diana (2023). Genotype by environment analysis on multi-canopy cropping system towards harvest in soybean. Heliyon.

[46] Gou H., Gan J., Liu J., Deng S., Gan L., Wang X., Zhao J., Xing H., Guo N. (2024). The GmCYP2-GmHAL3 module regulates salt tolerance in soybean seedlings. Environ. Exp. Bot..

[47] Cober E., Curtis D., Stewart D., Morrison M. (2014). Quantifying the effects of photoperiod, temperature and daily irradiance on flowering time of soybean isolines. Plants.

[48] Zhang L., Liu W., Tsegaw M., Xu X., Qi Y., Sapey E., Liu L., Wu T., Sun S., Han T. (2020). Principles and practices of the photo-thermal adaptability improvement in soybean. J. Integr. Agric..

[49] Fang Y., Wang L., Sapey E., Fu S., Wu T., Zeng H., Sun X., Qian S., Abdul M., Yuan S., Wu C., Hou W., Sun S., Han T. (2021). Speed-breeding system in soybean: integrating off-site generation advancement, fresh seeding, and marker-assisted selection. Front. Plant Sci..

[50] Fedorina J.V., Хлесткина Е.К., Сеферова И.В., Vishnyakova M.A. (2022). Genetic mechanisms underlying the expansion of soybean *Glycine max* (L.) Merr. cultivation to the north. Ecological Genetics.

