# Supplementary material for: Assessment of the genetic parameters of soybean genotypes for precocity and productivity in the various cultivation conditions
Source: Heliyon. 2024 Aug 13;10(16):e36135. doi: 10.1016/j.heliyon.2024.e36135 (PMC11367494; doi:10.1016/j.heliyon.2024.e36135)
Supplement: Multimedia component 1 [file mmc1.docx]

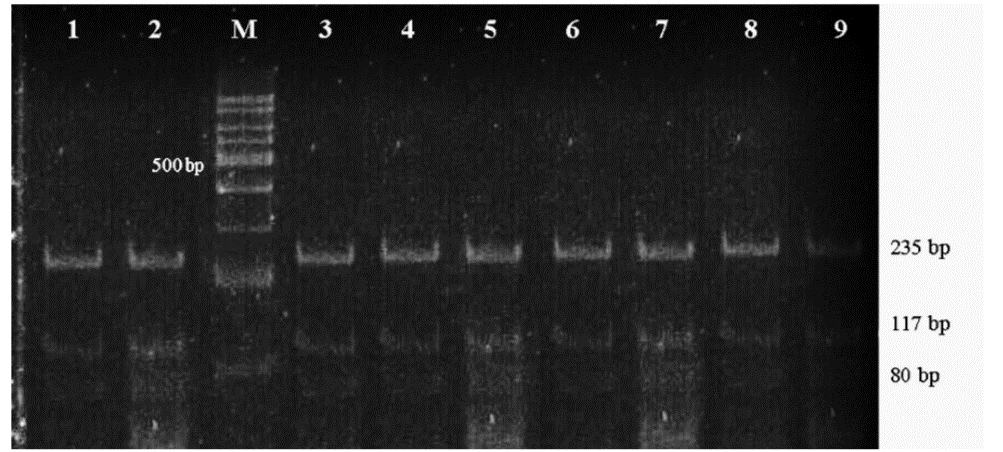


|  |
| --- |
| Fig. 1 – Electropherogram of soybean samples using marker E1_HinfI. М – marker (BioLabs, London, UK) 1- Suinong 10; 2 - Kendou 41; 3 - Beidou 19; 4 - Juisan 14-99; 5 - Jinyaan 55; 6 - Kenfeng 20; 7 - Kendou 68; 8 - Beidou 40; 9 -Beidou 36 |


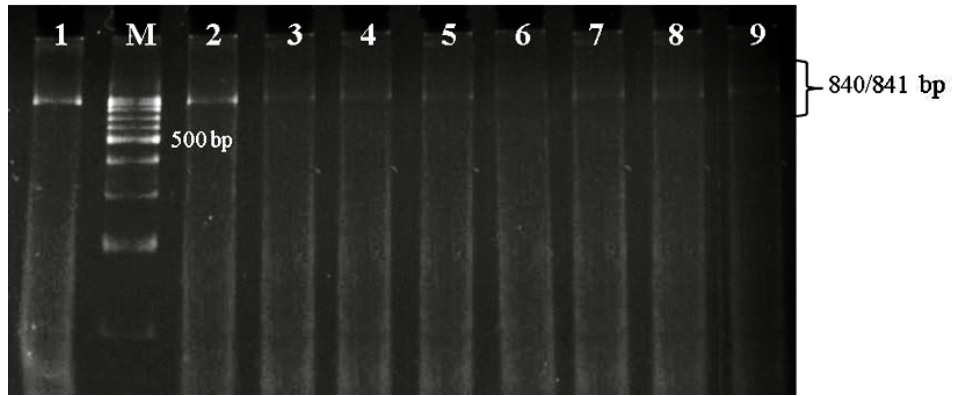


|  |
| --- |
| Fig. 2. Electropherogram of soybean samples using marker e1-re_STS.  М – marker; 1- Line 16, 2- Line 75, 3 –Line 113, 4- Line 90, 5 – Line 7, 6- Line 114, 7-Line 5, 8 – Line 115, 9-Line 80 |


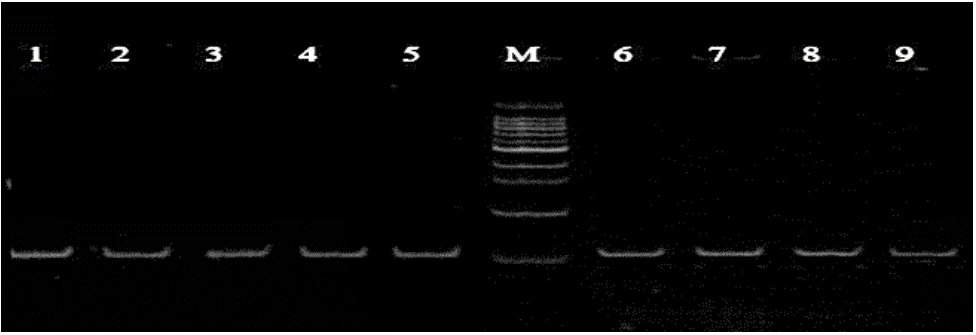


|  |
| --- |
| Fig. 3. Electropherogram of soybean samples using marker E2_DraI. М – marker; 1- Beidou 52; 2 - SK Doka; 3 - LongKen 310; 4 - Kenfong 21; 5 - Bara St; 6 – Ivushka St; 7 - Beidou 41; 8 - Kendou 61; 9 - Kenfeng 14 |


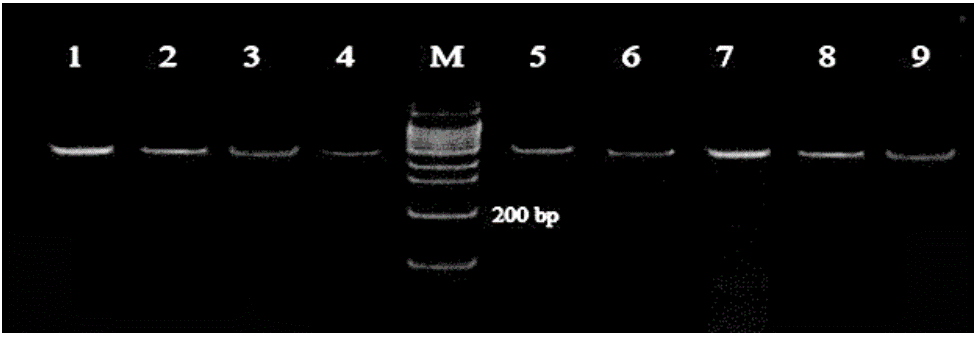


|  |
| --- |
| Fig. 4. Electropherogram of soybean samples using the E2_InDel marker. М – marker; 1- Beidou 52; 2 - SK Doka; 3 - LongKen 310; 4 - Kenfong 21; 5 - Bara St; 6 - Ivushka St; 7 - Beidou 41; 8 - Kendou 61; 9 -Kenfeng 14 |

| 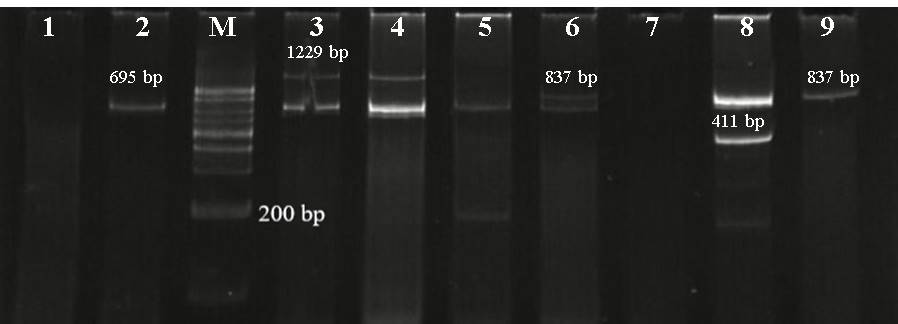 |
| --- |
| Fig. 5. s. Electropherogram of soybean samples using a marker *E*3*E*4_Mix. М – marker; 1- Line К-0115; 2 – Line К-0117; 3-Line К-0118, 4 – Line К-0119; 5 – Line К-0121; 6 – Line К-0121; 7 – Line К-0122; 8 – Line К-0123; 9 – Line К-0124 |
